# Bee Venom Acupuncture in the Treatment of Musculoskeletal System Disorders: A Comprehensive Review

**DOI:** 10.1155/prm/1766393

**Published:** 2025-12-26

**Authors:** Xiaodi Zou, Yanzhao Dong, Ahmad Alhaskawi, Haiying Zhou, Sohaib Hasan Abdullah Ezzi, Vishnu Goutham Kota, Mohamed Hasan Abdulla Hasan Abdulla, Sahar Ahmed Abdalbary, Zhenfeng Liu, Changxin Wang, Hui Lu

**Affiliations:** ^1^ Department of Orthopedics, The First Affiliated Hospital, Zhejiang University, Hangzhou City, Zhejiang Province, China, zju.edu.cn; ^2^ Department of Orthopedics, The Second Affiliated Hospital of Zhejiang Chinese Medical University, Hangzhou City, Zhejiang Province, China, z2hospital.com; ^3^ Department of Orthopedics, Third Xiangya Hospital, Central South University, Changsha City, Hunan Province, China, csu.edu.cn; ^4^ School of Medicine, Zhejiang University, Hangzhou City, Zhejiang Province, China, zju.edu.cn; ^5^ Department of Orthopedic Physical Therapy, Nahda University in Beni Suef, Beni Suef City, Beni Suef Governorate Province, Egypt, nahdauniversity.org; ^6^ Department of Nuclear Medicine, The First Affiliated Hospital, Zhejiang University, Hangzhou City, Zhejiang Province, China, zju.edu.cn

**Keywords:** bee venom acupuncture, complementary therapies, melittin, musculoskeletal disorders, pain management

## Abstract

Disorders of the musculoskeletal system cover a broad spectrum of conditions that impact the muscles, skeletal structure, joints, tendons, and ligaments, leading to discomfort, swelling, and limited movement. Bee venom acupuncture (BVA), a practice commonly used in Asian countries, has been employed for a considerable time in traditional medicine systems to treat these disorders by utilizing bee venom and its main constituent, melittin. The purpose of this extensive evaluation on is to offer a detailed examination of the healing capabilities, modes of operation medical uses, and safety records of melittin and BVA in the treatment of different musculoskeletal disorders.

## 1. Introduction

The high occurrence and resulting pain and disability of musculoskeletal disorders place a substantial strain on healthcare systems worldwide [1].These disorders may be congenital and acquired and may include deformities, amputations, or other abnormalities. Musculoskeletal disorders primarily consist of back and neck pain disorders, arthritic conditions, and soft tissues syndromes affecting the tendons, ligaments, muscles, and cartilages [1].

**Figure 1:**
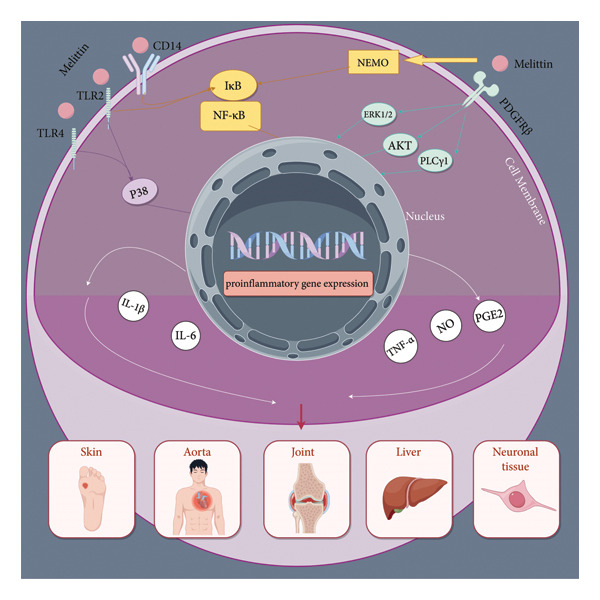
Graphical abstract.

Industrialized nations have a particularly high prevalence of musculoskeletal disorders, which are the primary cause of pain and disability worldwide [1–4]. As per the WHO report, they also make up the largest proportion of individuals requiring rehabilitation worldwide, constituting around 66% of all adults in need of rehabilitations. NSAIDs, such as naproxen, ibuprofen, and diclofenac, are the most frequently prescribed medicines for musculoskeletal disorders [5]. So far, only a limited number of therapy options have been developed [6]. Bee venom acupuncture (BVA), a traditional Asian medical practice primarily from Korea and China, utilizes bee venom injection into specific acupuncture points to treat arthritis, alleviate pain, and address rheumatoid diseases [7, 8] (Figure 1).

Peptides (such as melittin, adolapin, and apamin), enzymes like phospholipase *A*_2_, and amines are all examples of BV compounds. The broad spectrum of pharmacological properties and potential therapeutic benefits of melittin, a crucial element found in bee venom, has generated considerable attention in the field of musculoskeletal disorder treatment. With its anti‐inflammatory, pain‐relieving, anti‐arthritis, antifibrotic characteristics [9], this substance shows potential as a viable option in the treatment of ailments like rheumatoid arthritis, osteoarthritis, tendinitis, and myofascial pain syndromes.

## 2. Mechanisms

The migration of immune cells toward the site of damaged tissues and the release of compounds trigger an inflammatory reaction, which is a standard response to tissue harm, pathogen‐induced infection, and chemical stimulation [10]. Stimulation of macrophages occurs when exposed to proinflammatory agents like lipopolysaccharide (LPS), causing the activation of TLR4 receptors. Subsequently, these receptors prompt the initiation of cytokine generation via pathways involved in inflammatory signaling, such as NF‐kB signaling [11, 12].

Oxidative stress and molecular damage, caused by reactive oxygen species (ROS) like O_2_, H_2_O_2_, OH, and NO, result in inflammatory diseases [13, 14]. Hence, in order to achieve an anti‐inflammatory outcome, it is imperative to suppress intracellular ROS, and it is vital to discover natural substances that possess antioxidant and anti‐inflammatory characteristics.

Melittin has the capacity to regulate the generation and secretion of various cytokines, which are compact proteins involved in cellular communication during inflammatory reactions. Research has shown that it can hinder the generation of proinflammatory cytokines like TNF‐*α* and IL‐1*β* [15]. Moreover, it has the potential to affect the secretion of neuropeptides, which are tiny chemical messengers implicated in the transmission of pain sensations. In particular, melittin has demonstrated its ability to impede the secretion of substance *p*, a neuropeptide accountable for the cognitive recognition of pain, from sensory neurons’ terminals [15]. In addition, melittin has the ability to control various signaling pathways linked to inflammation and pain. It has been observed that the inhibition of nuclear factor kappa‐B (NF‐*κ*B) [12], a transcription factor responsible for regulating the expression of numerous proinflammatory genes, has been documented. Moreover, melittin possesses the capability to activate the adenosine monophosphate‐activated protein kinase (AMPK) pathway, thereby leading to the manifestation of anti‐inflammatory and pain‐alleviating effects [15].

To summarize, these mechanisms ultimately lead to the discharge of proinflammatory substances like inflammatory cytokines, TNF, NO, or PGE2 into the extracellular media or blood vessels. The inflammatory effects on tissues caused by these chemicals indicate that melittin’s ability to inhibit their formation suggests an anti‐inflammatory role (Figure 1) [16].

Additionally, some studies have investigated the underlying mechanisms in specific diseases. In inflammatory pain, BVA (*A. dorsata*, oral, 2.0 mg/kg) has been shown to reduce IL‐6 and TNF‐*α* levels in a collagen Type II–induced arthritis pain model [17]. Additionally, BVA (*A. mellifera*, dorsal, 0.25 mg/kg) normalizes TNF‐*α* independently of methotrexate and restores enzyme activity (APN, DPPIV) in synovial fluid and PBMCs [18]. Furthermore, BVA (*A. mellifera*, ST36 acupoint, 0.8 mg/kg) mitigates spinal astrocyte activation via *α*‐2A and *α*‐2C adrenoceptor pathways, resulting in potent analgesic effects [19].

For neuropathic pain, BVA (*A. mellifera*, GB39, LI4, LV3, SJ5 acupoints, 0.1 mg/mL) mimics the action of norepinephrine reuptake inhibitors in spinal nerve injury models [20]. Combination therapy with BVA (ST36 acupoint, 1.0 mg/kg) and venlafaxine (oral, 40 mg/kg) effectively reduces paclitaxel‐induced allodynia through 5‐HT1/5‐HT2, 5‐HT3, and *α*2‐adrenergic receptors [21].

In cases of mechanical allodynia, BVA (ST36 acupoint, 1.0 mg/kg) exerts potent suppressive effects in paclitaxel‐induced allodynia mediated by spinal *α*2‐adrenergic receptors [22]. Repetitive BVA injections (ST36 acupoint, 0.25 mg/kg) decrease oxaliplatin‐induced mechanical allodynia through *α*2‐adrenoceptor mechanisms and recovery of intraepidermal nerve fibers (IENFs) [23].

Regarding cold allodynia, BVA (GV3 acupoint, 0.25 mg/kg) alleviates oxaliplatin‐induced cold allodynia through spinal 5‐HT3 receptor activation [24]. The antiallodynic effects of BVA (ST36 acupoint, 2.5 mg/kg) in chronic constrictive injury models are mediated by spinal *α*2‐adrenoceptors [25].

In postischemic pain, BVA (1.0 mg/kg, hind paw) reduces neurokinin type 1 (NK‐1) receptor expression in DRG, leading to alleviation of pain symptoms [26].

**Figure 2:**
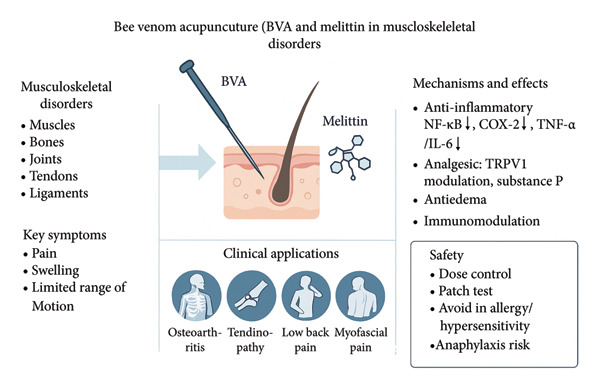
The primary mechanisms through which melittin exerts its anti‐inflammatory effects. Specifically, melittin acts by suppressing the signal pathways associated with Toll‐like receptors 2 and 4, cluster of differentiation 14, nuclear factor kappa‐B essential modulator, and platelet‐derived growth factor receptor *β*. This inhibition leads to a decrease in the activation of p38, extracellular signal‐regulated kinases 1 and 2, protein kinase B, phospholipase Cγ1, and the translocation of nuclear factor kappa‐B into the nucleus. Consequently, this inhibition results in a reduction of inflammation in various tissues, including the skin, aorta, joints, liver, and neuronal tissue.

For prostatic pain, melittin (0.05 mg, prostate lobes) suppresses COX‐2 expression, effectively alleviating CFA‐induced prostatitis pain (Figure 2) [26].

### 2.1. Preclinical Studies

Understanding the pharmacological properties, toxicity, and biological effects of melittin and BVA through preclinical studies is essential in order to facilitate their translation into clinical therapies. Melittin, a primary constituent of bee venom, has exhibited anti‐inflammatory, pain‐relieving, antioxidant, and antimicrobial characteristics. Additionally, it has shown anticancer effects in preclinical models [27, 28]. Nevertheless, the use of this treatment in medical practice has been restricted because of concerns regarding its toxicity and lack of specificity, degradation, inefficient systemic delivery, and limited bioavailability [28]. In order to overcome these constraints, different nanocarrier platforms have been created with the aim of enhancing the stability of melittin, minimizing adverse reactions, and improving its targeted delivery to tumors. These systems include liposomes, positively charged polymers, and lipodisks [29]**.**

Several studies suggest that acupuncture may enhance the outcomes of BV treatment, assisting in the attainment of therapeutic goals. Research and a review conducted by Dr. Lee’s team have demonstrated the effectiveness of BVA in alleviating symptoms of RA and OA. To examine the potential site specificity, a series of comparative experiments were carried out using BVA. These experiments aimed to compare the impacts of stimulation on both acupoints and non‐acupoints in an animal model of RA induced by adjuvants [30, 31]. In a chronic arthritis animal model, injecting BV directly into acupoint St36 (also called Zusanli) produced a potent pain‐relieving effect compared to injecting it into a non‐acupoint. This indicates that utilizing BV for acupoint stimulation offers a viable approach for pain management. In the rat arthritis model induced by Type II collagen, the study demonstrated the efficacy of BVA in acupoints BL23 and ST36. Furthermore, the research revealed that BVA reduced the levels of ROS and the activity of proteolytic enzymes, thereby decreasing the risk of oxidative damage to the synovial fluid.

Compared with alternative therapy methods, the application of BVA at acupoint BL23 significantly decreased the levels of proteolytic enzymes and the extent of oxidative damage caused by ROS to the proteins in synovial fluid. Furthermore, the application of BVA on acupoint BL23 exhibited more remarkable anti‐inflammatory properties in rats with carrageenan‐induced knee arthritis.

In mature Sprague Dawley rats, the application of BVA on acupoint ST36 resulted in a significant decrease in the duration of paw‐licking during the later stage of the formalin test. Moreover, it effectively suppressed the activation of Fos in the spinal cord induced by the injection of formalin [30, 32]. In contrast, this was in relation to a random non‐acupoint situated on the posterior side. This implies that the outcomes of BVA are influenced by the locations of the injections, as injecting into an acupoint produces significantly more noticeable effects in comparison to injecting into a non‐acupoint. In a CIA model induced in mice using Type II collagen, Lee et al. [33] observed that administering acupuncture at a point (Zusanli) near both knees, twice a week for a total of five sessions, led to a significant reduction in the occurrence of arthritis, the average arthritis score, and the count of affected limbs when compared to a control group.

In this study, the BV group exhibited a reduction in the production of TNF‐α compared to the control group among the proinflammatory cytokines present in the serum. After undergoing BVA treatment, the examination of the joints in mice with Type II CIA showed a decrease in inflammatory symptoms and a reduction in the infiltration of lymphocytes.

Rodent models, including mice and rats, have been widely used in in vivo research (Table 1). These models have played a crucial role in investigating the therapeutic effects of BV. BV has been the subject of recent research, which has emphasized its positive effects on a range of conditions, such as Alzheimer’s disease (AD) [34], chronic prostatitis [26], gouty arthritis [35], and amyotrophic lateral sclerosis (ALS) [36]. Furthermore, studies involving rabbits have also been conducted [37] (Table 1).

**Table 1:** Preclinical studies related to their animal models.

Disease model	Animals	Interventions	Manifestation	Adverse events
Arthritis pain–FCA and collagen II [17]	Male Wistar rats (190–210 g); E1 *n* = 6, E2 *n* = 6	E1: FCA + D2:D64D3:D42:E5D2:F61E6 rats, BV injected once (right hind paw) 1 mg/200 g; E2: Collagen II rats, BV injected once 1 mg/200 g; AC1: FCA rats, indomethacin 2.0 mg/kg (oral); AC2: Collagen II rats, indomethacin 2.0 mg/kg (oral); NC1/NC2: saline 0.1 mL	Dorsal flexion pain score improved: E1 versus NC1 *p* < 0.05 (day 21), *p* < 0.01 (Day 28); E2 versus NC2 *p* < 0.05 (Days 27 and 36).	Not reported
Arthritis pain–Collagen II [18]	Male Wistar rats (150–160 g); E1 *n* = 5, E2 *n* = 3	E1: BV s.c. once (dorsal), 0.25 mg/kg in 50 μL; E2: E1 + methotrexate s.c. 0.3 mg/kg in 300 μL; AC1: Saline (50 μL); AC2: methotrexate 0.3 mg/kg; NC: healthy rats + saline.	Mechanical threshold of hyperalgesia: BV alone showed no significant change versus NC; MTX alone and BV + MTX decreased hyperalgesia versus NC.	Not reported
Inflammatory pain—formalin test (preinjection) [19]	Male ICR mice (20–25 g); E1 *n* = 8, E2 *n* = 8	E1: BV s.c. at ST36 before formalin, 0.8 mg/kg in 20 μL; E2: BV s.c. at ST36 before formalin, 0.08 mg/kg in 20 μL; NC: saline 20 μL at ST36.	Paw‐licking time: E1 versus NC NS (first 10 min), *p* < 0.001 (late 20 min); E2 versus NC NS (early and late).	Not reported
Inflammatory pain—formalin test (postinjection + adrenergic modulation) [38]	Male ICR mice (25–30 g); E *n* = 8, AC1 *n* = 8, AC2 *n* = 8	E: BV s.c. at ST36 after formalin, 0.8 mg/kg (1:1000 dilution), 100 μL; AC1: BV at ST36 + hydroxydopamine i.p. 100 μL; AC2: BV at ST36 + epinephrine i.p. 100 μL; NC: saline 100 μL at ST36.	Paw‐licking time (late phase): E versus NC *p* < 0.001; AC1 versus NC *p* < 0.001; E versus AC1 NS; E versus AC2 *p* < 0.01.	Not reported
Osteoarthritis pain—Collagen II [39]	Male Sprague Dawley rats (200–250 g); E1 *n* = 10, E2 *n* = 10, AC *n* = 10	E1: BV s.c. at ST36, 1.0 mg/kg (once); E2: BV s.c. at ST36, 2.0 mg/kg; AC: BV i.p., 1.0 mg/kg.	Tail‐flick latency increased: E1 versus AC *p* < 0.05 (10 min), *p* < 0.01 (20), *p* < 0.001 (30 and 45), *p* < 0.01 (60). E1 versus E2 significant at 10–60 min.	Not reported

### 2.2. Clinical Applications

Given the various biological functions and regulatory processes of BV, it is unsurprising that its use in medical treatment is attracting interest. Although the beneficial effects of BV in reducing inflammation, preventing cell death, inhibiting fibrosis, and preventing atherosclerosis have been recognized for a while, its potential impact on cardiovascular and neurological conditions has only recently come to light [40].

### 2.3. Shoulder Pain

The systematic review analyzed seven randomized controlled trials (RCTs) [41] and found that the group receiving BVA (administered at acupoints GB21, LI11, LI15, LI16, SI3, SI9, SI10, SI11, TE14, and UE12) in addition to conventional treatment (CT) experienced a significant reduction in shoulder pain, as opposed to the group receiving saline injection plus CT. The distinction was apparent in both the pain rating scale (PRS) (*p* = 0.009) and the visual analog scale (VAS) (*p* = 0.03). According to the results of this research, incorporating BVA into CT may be regarded as a viable alternative for alleviating shoulder unease. Nevertheless, it is crucial to acknowledge that the uniformity of dosage and frequency of treatment must be meticulously deliberated and executed in order to guarantee the best outcomes. Additional investigation and medical recommendations are necessary to establish precise procedures for utilizing BVA alongside CT in the management of shoulder discomfort.

The effectiveness of BVA in treating pain related to adhesive capsulitis was assessed in two RCTs [42, 43]. In a particular study [43], participants were categorized into three groups: BVA Group 1, which was administered 0.1 mg/mL BVA in addition to physical therapy; BVA Group 2, which was given 0.03 mg/mL BVA alongside physical therapy; and the control group, which received a saline injection along with physical therapy. BV was injected into acupoints GB21, LI15, LI16, SI11, and TE14 in both BVA groups. The BVA Group 1 showed notable enhancements in terms of the shoulder pain and disability index (SPADI) at 8 days (*p* = 0.025) and 12 days (*p* = 0.014), the VAS score at rest during the eighth week (*p* = 0.048), and the motion score during the 12th week (*p* = 0.029) when compared to the control group.

Group 1 of the BVA team underwent a total of 16 therapy sessions, with a BV size of 14.8 mL. Furthermore, a telephonic survey was carried out as a 1‐year follow‐up investigation, utilizing telephonic interviews as per a previously published RCT [42]. At the 1‐year follow‐up, the SPADI scores showed a notable disparity between the group that received 0.1 mg/mL BVA alongside physiotherapy and the group that received saline injection in addition to physiotherapy (*p* = 0.043), indicating a significant statistical distinction. The results partially validated the therapeutic benefits of BVA in treating pain caused by adhesive capsulitis, especially when given at a concentration of 0.1 mg/mL instead of 0.03 mg/mL. Furthermore, there were also some minor adverse events (AEs) that were observed, which further emphasized the safety profile of BVA treatment.

### 2.4. Back Pain

The efficacy of BVA in treating low back pain was investigated in two RCTs [44, 45] and one retrospective study [46]. In an RCT conducted by Seo et al. [44], a total of 54 individuals received either BVA (administered at acupoints BL23, BL24, BL25, GB30, GV3, GV4, and GV5) along with NSAIDs, or saline injection plus NSAIDs. The treatment consisted of six sessions over a span of 3 weeks. The BVA plus NSAID group exhibited a significantly reduced level of chronic low back pain (*p* = 0.0486) compared to the control group. Throughout the six treatment sessions, a total volume of 28 mL of BVA with a concentration of 0.05 mg/mL was administered. In another study by Shin et al. [45], a total of 60 patients were randomly assigned to receive either saline injections or BVA (administered at acupoints BL23, BL24, and BL25) for the treatment of low back pain. The BVA group showed a statistically significant difference (*p* = 0.0087) compared to the saline injection group in terms of pain intensity measured using the VAS. The total number of treatment sessions was eight, with a BVA concentration of 0.05 mg/mL and a total volume of 4.8 mL.

A total of 524 participants who received nonsurgical complementary and alternative medicine treatments, including BVA, acupuncture, herbal medicine, and chuna, for low back pain associated with intervertebral disc herniation were included in the study. The treatment involved the administration of these therapies at four to five acupoints around the lumbar spine. The participants reported a mean numeral rating scale (NRS) score ranging from 3.18 to 2.29 (95% confidence interval [CI], 2.99–3.38), indicating a reduction in pain intensity. The average number of treatment sessions was 2.3, with a standard deviation of 1.8. The BVA concentration used in the treatment was reported to be 0.1 mg/mL, while the total volume administered was not specified. During the course of treatment, eight patients experienced allergic reactions attributed to BVA. The clinical trial conducted by Shin et al. [46] does not explicitly focus on investigating BVA as a treatment for low back pain, although it does provide some evidence supporting its efficacy. It is worth noting that the concentration of BVA used in both RCTs by Seo et al. [44] and Shin et al. [45] was consistent at 0.05 mg/mL. However, there was a significant difference in the overall volume administered between the two studies, highlighting the need for standardization in terms of therapy sessions and dosage. Standardizing these factors would help ensure consistency and comparability across studies, enabling a more accurate assessment of the effectiveness of BVA as a treatment for low back pain.

### 2.5. Inflammatory Pain

A research study was conducted with 361 individuals experiencing knee osteoarthritis pain [47]. These participants were randomly divided into two groups, one receiving histamine injections and the other receiving BVA. The BVA team was administered BV injections at particular acupoints, which comprised BL40, BL19, BL21, BL23, BL25, BL27, and ST34. In total, each knee (knee top) received five injections, along with one injection on the medial side and two injections on the lateral side. Both the BVA group and the control group were administered the same acupoint injections. Following a 12‐week treatment period, the BVA group exhibited a noteworthy decrease in their levels of pain, as indicated by the WOMAC pain score (95% CI, 0.3–2.0, *p* = 0.0010). During the 12 treatment sessions, a combination of 1 mg of BV powder and 1 mL of 0.5% lidocaine was administered as a mixture, with a total volume of 17.1 mL.

A total of 60 patients with knee osteoarthritis were enrolled in the study [48] and randomly divided into two groups: one group received traditional acupuncture treatment (20 patients) and the other group received BVA (40 patients). The treatment regimen consisted of two sessions per week for 4 weeks. The traditional acupuncture group was treated with stainless steel needles, whereas the BVA group received injections of bee venom diluted in normal saline, at a concentration of 0.03 mg/mL. Each acupoint was injected with 0.1 mL of the solution, with a total injection volume not exceeding 1 mL. The results showed that the BVA group had a significantly higher mean treatment efficacy score (3.20 ± 0.72) compared to the traditional acupuncture group (2.55 ± 0.67, *p* < 0.01), indicating superior effectiveness of BVA in pain relief. Moreover, BVA demonstrated favorable therapeutic effects across different disease stages (acute, subacute, chronic), lesion laterality (unilateral or bilateral), and radiological grades. Infrared thermography (IRT) assessment also confirmed the therapeutic efficacy of BVA, aligning with the subjective pain relief scores.

### 2.6. Poststroke Pain

According to a meta‐analysis of two RCTs [49], it was found that the BVA group experienced a significant decrease in poststroke shoulder pain compared to the saline injection group. This reduction was measured using the VAS, and the difference was statistically significant (*p* = 0.02). The BVA team underwent therapy at acupoints EX‐UE70, GB21, LI11, LI15, SI3, SI9, SI10, SI11, and TE14. BVA concentration varied between 0.01 and 0.5 mg per milliliter. The number of treatment sessions ranged from 6 to 12, while the administered BVA volume varied between 0.9 mL and 13.5 mL. A separate single‐blind RCT42 examined the effectiveness of BVA compared with saline for individuals experiencing central poststroke pain. BVA or saline was given at the acupoints GB21, GB31, GB39, LI11, LI15, and ST36. Further details regarding the treatment concentration, total sessions, and volume were not provided in the given context. Following a treatment period of 3 weeks, the BVA group demonstrated a higher augmentation in the VAS rating in contrast to the control group (*p* = 0.009). In the study, it was reported that a total of 1.8 mL of BV was administered during six treatment sessions, but no information about the exact concentration used was given. It is worth mentioning that there were no reported negative incidents in the BVA group. The results indicate that BVA shows efficacy in alleviating musculoskeletal pain and pain experienced after a stroke.

### 2.7. Complex Regional Pain Syndrome

After undergoing toe surgery, a patient diagnosed with complex regional pain syndrome received BVA treatment at the GB43 acupoint [50]. The patient experienced a significant improvement in pain levels as assessed by the NRS. The worst level of pain decreased from 8 to 0, the average level from 5 to 0, and the best level from 3 to 0 after the BVA treatment. The treatment consisted of a total of 14 sessions, with a volume of 4.55 mL of BVA administered. However, the specific BVA content used in the treatment was not disclosed. Notably, no BVA‐related side effects were reported during the course of treatment.

### 2.8. Parkinson’s Disease

The neuroprotective effect and the suppression of microglial activation of BVA have been demonstrated in animal models [51]. In a clinical trial [52], a total of 43 patients with Parkinson’s disease were recruited. They were randomly assigned to one of three groups: acupuncture, BVA, or no treatment. Participants in the treatment groups received stimulation with acupuncture or BVA at 10 acupuncture points (bilateral GB 20, LI 11, GB 34, ST36, and LR 3) twice a week for 8 weeks (16 total sessions). The results showed that the BVA group had significant improvements in the total score of Unified Parkinson’s Disease Rating Scale (UPDRS) and some parts of II and III, the Berg Balance Scale, and the 30‐m walking time.

### 2.9. Acute Ankle Sprain

A clinical study [53] involved 32 patients diagnosed with acute ankle sprain. These patients were randomly allocated into two groups: one group received traditional acupuncture, while the other group received BVA (0.5 mg/L, twice a week). The result showed that bee venom therapy demonstrated faster effects in reducing pain, decreasing swelling, and improving the ROM. Especially in the first to fourth weeks after treatment, it was superior to ordinary acupuncture therapy in terms of the improvement of the VAS score, swelling degree, and ROM of joints.

### 2.10. Soft Tissue Damage in Neck

A clinical study [54] recruited a total of 34 patients. All patients presented with simple soft tissue injuries of the neck without any structural cervical spine abnormalities or neurological deficits. The participants were randomly assigned into two equal groups (*n* = 17 each): Group A received Korean bee venom therapy, while Group B underwent conventional acupuncture therapy.

In Group A, a diluted bee venom solution at a ratio of 10,000:1 was administered at a dosage of 0.02–0.03 cc per acupoint, twice per week. The selected acupoints included Ashi points and several distal points: GB20, GV14, GB21, SI11, SI9, LI11, TE5, LI4, TE3, and SI3. Group B received standard acupuncture therapy using 0.25 × 25 mm stainless steel needles, also administered twice weekly.

The results showed that Group A experienced a more substantial reduction in pain and greater improvement in ROM compared to Group B. Specifically, the VAS score in Group A decreased from 7.2 ± 0.44 before treatment to 3.6 ± 0.44 after the first session, and further to 1.5 ± 0.36 after the second session. In contrast, Group B’s VAS score dropped from 6.4 ± 0.46 to 4.9 ± 0.52 after the first session and to 4.0 ± 0.56 after the second session. The difference in VAS scores between the groups was statistically significant after both the first and second treatments (*p* < 0.01).

Regarding ROM, Group A showed improvement from 37.7 ± 3.98° at baseline to 50.6 ± 2.73° after the first session and 55.8 ± 2.12° after the second session. In Group B, ROM increased from 36.5 ± 4.30° to 41.2 ± 3.42° after the first session and to 46.8 ± 3.65° after the second session. Again, the improvements in Group A were significantly greater than those in Group B at both time points (*p* < 0.01).

### 2.11. Lateral Epicondylitis

A total of 24 patients were enrolled in the study [55] and randomly divided into two groups, with 12 patients in each group. The bee venom therapy group received diluted bee venom at a concentration of 0.05 mg/mL, with an injection volume of 0.1 cc–0.4 cc per acupoint. Treatments were administered every other day, for a total of six sessions. The traditional acupuncture group was treated using stainless steel needles measuring 0.25 × 30 mm, with a needle retention time of 20 min. Treatments were also administered every other day for six sessions. Selected acupoints included Ashi points, LI11, and LI10, among others.

In terms of pain assessment, the VAS score in the bee venom therapy group significantly decreased from 5.08 ± 1.26 before treatment to 4.00 ± 0.91 at 2 weeks (*p* < 0.05), 2.83 ± 0.90 at 4 weeks (*p* < 0.05), and 1.42 ± 0.76 at 6 weeks (*p* < 0.05). In the traditional acupuncture group, the VAS score decreased from 5.17 ± 1.07 before treatment to 4.42 ± 0.86 at 2 weeks (*p* < 0.05), 3.58 ± 0.76 at 4 weeks (*p* < 0.05), and 2.33 ± 0.75 at 6 weeks (*p* < 0.05). Comparison between the two groups showed that the bee venom therapy group experienced significantly greater pain relief at both 4 and 6 weeks post‐treatment (*p* < 0.05).

In terms of grip strength assessment, the bee venom therapy group showed a significant increase from 24.99 ± 6.20 before treatment to 25.89 ± 5.70 at 2 weeks (*p* < 0.05), 27.09 ± 6.12 at 4 weeks (*p* < 0.05), and 29.43 ± 5.61 at 6 weeks (*p* < 0.05). The traditional acupuncture group showed an increase from 24.25 ± 6.17 before treatment to 25.27 ± 6.23 at 2 weeks (*p* < 0.05), 26.69 ± 5.82 at 4 weeks (*p* < 0.05), and 28.17 ± 5.06 at 6 weeks (*p* < 0.05). Both groups demonstrated significant improvements in grip strength, with no significant difference between the two groups (*p* > 0.05) (Table 2).

**Table 2:** Clinical application of bee venom for musculoskeletal pain.

Disease model	Animals	Interventions	Manifestation	Adverse events
Shoulder pain—systematic review [41]	Human, systematic review (*n* = 173)	BVA: acupoints GB21, LI11, LI15, LI16, SI3, SI9, SI10, SI11, TE14, EX‐UE12; concentration 0.03–0.5 mg/mL (saline); per‐session 0.1–1.5 mL; total 6–16 sessions (0.6–14.8 mL). Comparator: saline injection at the same acupoints/sessions/dose.	BVA + conventional therapy versus saline + CT: significant improvement in VAS (*p* = 0.03) and pain rating scale (PRS) (*p* = 0.009).	Pain 2; pruritus 8; burning 3; pruritus/local swelling/redness 30; mild generalized swelling/aching 1.
Myofascial shoulder pain—RCT [56]	Human, RCT (*n* = 50)	BVA: GB21; conc. NR; 1 session 1 mL; total 2 sessions (2 mL). Needle‐free BVA: GB21; conc. NR; 1 session 1 mL; total 2 sessions (2 mL).	VAS improved significantly in both groups (*p* values NR).	BVA: Fatigue 1, purpura 1, edema 2, headache 1, cold 1, itching 16, redness 14. Needle‐free BVA: Inflammation 1, purpura 2, headache 2, redness 1.
Adhesive capsulitis pain—RCT [43]	Human, RCT (BVA(a) *n* = 22/BVA(b) *n* = 23)	BVA group1 (a): GB21, LI15, LI16, SI11, TE14; 0.1 mg/mL; visits 1–3: 0.4/0.6/0.8 mL; visits 4–16: 1 mL; total 16 sessions 14.8 mL. BVA Group 2 (b): Same acupoints; 0.03 mg/mL; volume schedule same; total 14.8 mL. Comparator: saline injection with same acupoints/schedule/dose.	BVA Group 1 + PT versus saline + PT: SPADI improved at Day 8 (*p* = 0.025) and Day 12 (*p* = 0.014); VAS at rest at Week 8 (*p* = 0.048) and during motion at Week 12 (*p* = 0.029). BVA Group 1 + PT versus BVA Group 2 + PT: NS.	BVA (a)/BVA (b): slight pruritus/local swelling/redness 30; BVA (a): mild generalized swelling/aching 1.
Adhesive capsulitis pain—1‐year follow‐up of RCT [42]	Human, follow‐up of prior RCT [16] (BVA (a) *n* = 20/BVA (b) *n* = 22)	BVA Group 1 (a): GB21, LI15, LI16, SI11, TE14; 0.1 mg/mL; total 16 sessions 14.8 mL. BVA Group 2 (b): GB21, LI15, LI16, SI11, TE14; 0.03 mg/mL; total 16 sessions 14.8 mL. Comparator: saline injection same acupoints/sessions/dose.	BVA Group 1 + PT versus saline + PT: SPADI improved at 1 year (*p* = 0.043). Pain VRS among 3 groups: NS.	Not reported
Chronic low back pain—RCT (NSAID co‐treatment) [44]	Human, RCT (*n* = 27)	BVA: BL23, BL24, BL25, GB30, GV3, GV4, GV5; 0.05 mg/mL (saline); per‐session volume 2 mL (wk1), 4 mL (wk2), 8 mL (wk3); total 6 sessions (28 mL). Comparator: saline injection; both groups received NSAIDs.	BVA + NSAID versus saline + NSAID: significant VAS reduction (*p* = 0.0486).	Itching/sensation 4, headache 1, generalized myalgia 1.
Chronic low back pain—RCT [45]	Human, RCT (*n* = 60)	BVA: BL23, BL24, BL25; 0.05 mg/mL (saline); per‐session 0.6 mL; total 8 sessions (4.8 mL). Comparator: saline injection (same acupoints/sessions/dose).	BVA versus saline: significant reduction in VAS pain intensity (*p* = 0.0087).	Pruritus 15, skin flare 5, edema 4, skin rash 2.
Low back pain—retrospective study [46]	Human, retrospective (*n* = 524)	BVA: 4–5 acupoints around lumbar spine; 0.1 mg/mL (saline); per‐session 0.5–1 mL; mean total sessions 2.3 ± 1.8. Co‐interventions: herbal medicine + acupuncture + chuna.	Average NRS reduction 3.18–2.29 (95% CI 2.99–3.38).	Allergic reactions 8.
Knee osteoarthritis pain—clinical RCT [47]	Human, RCT (*n* = 361)	BVA: acupoints BL40, BL19, BL21, BL23, BL25, BL27, ST34; five on each knee (knee top), eye‐1 medial, eye‐2 lateral. Concentration: bee venom powder 1 mg + 1 mL 0.5% lidocaine mixed. Session volume: 1.2 mL (Weeks 1–3), 1.5 mL (Weeks 4–12); total 12 sessions, 17.1 mL. Comparator: Histamine injection 0.275 mg/mL at the same acupoints/sessions/dose.	BVA versus histamine: significant improvement in WOMAC pain score (*p* = 0.0010).	Total AEs 177; injection‐site AEs 15
Parkinson’s disease [52]	Human, RCT (*n* = 43)	**Acupuncture:** 10 standardized acupoints (bilateral GB20, LI11, GB34, ST36, LR3); stainless steel needles (0.25 × 30 mm), depth 1–1.5 cm, rotated 2 Hz for 10 s to elicit *Deqi*, retained 20 min. **Bee venom acupuncture (BVA):** 0.1 mL of 0.005% diluted bee venom injected at same points. **Control:** no treatment for 8 weeks, then crossover to active treatment.	Both acupuncture and bee venom acupuncture improved motor function (UPDRS III) and overall PD symptoms, with **BVA showing stronger and broader benefits** (balance and gait).	Not reported.

### 2.12. Safety and AEs

BVA is a therapeutic modality that combines principles of traditional acupuncture with the pharmacological effects of purified bee venom [57]. Unlike bee sting therapy (BST), which involves the direct use of live bee stings, BVA employs a controlled administration of diluted and sterilized bee venom extracted primarily from *Apis mellifera* worker bees [58]. This venom is typically injected subcutaneously or intramuscularly at specific acupuncture points corresponding to the meridians of traditional Chinese medicine, thereby aiming to harness both the mechanical stimulation of acupuncture and the biochemical actions of bee venom constituents [40].

Clinically, BVA has been explored for a range of musculoskeletal and neurological conditions, including rheumatoid arthritis, osteoarthritis, low back pain, and poststroke spasticity. The mechanism of action is hypothesized to involve modulation of inflammatory mediators, regulation of cytokine expression, and desensitization of pain receptors, such as TRPV1 and ASIC channels [59]. Moreover, the localized administration of venom at acupuncture points is thought to potentiate therapeutic efficacy by targeting areas with heightened physiological activity and neurovascular density.

Despite its potential benefits, BVA requires rigorous clinical oversight due to the inherent bioactivity of bee venom. Factors such as the degree of dilution, injection volume, site specificity, and individual susceptibility can all influence treatment outcomes and the risk of AEs. Therefore, adherence to standardized protocols, comprehensive patient screening, and post‐treatment monitoring are crucial to ensure both safety and therapeutic success.

Ensuring safety is of utmost importance, and the examination investigates the documented negative impacts and necessary precautions linked to the administration of BVA and melittin. The BVA therapy groups in the studies reported only minor side effects, including itching, a burning sensation, localized swelling, and soreness. However, no severe or life‐threatening reactions such as anaphylaxis were observed [60](Table 3).

**Table 3:** Adverse effects of BVA [22, 60, 61].

Minor side effects	Severe side effects
Itching	Anaphylaxis
Burning sensation	
Localized swelling	
Soreness	

### 2.13. Future Directions

Although numerous scholarly investigations have explored BV, its constituents, and their therapeutic applications, further research remains essential to clarify its mechanisms of action and metabolic pathways. Despite frequent references to BV components in the literature, the precise molecular targets, pharmacokinetics, and biotransformation processes are still inadequately understood. Hence, additional studies are needed to deepen our understanding of these aspects and elucidate how BV exerts its biological effects in vivo [40]. Furthermore, it is imperative to strengthen the evidence base regarding the safety profile of BVA. While most existing studies have reported only mild and transient adverse reactions—such as localized itching, erythema, or swelling—the potential for more serious hypersensitivity or anaphylactic responses cannot be disregarded, particularly when treatment is administered without standardized safety protocols. Therefore, future research should focus on establishing rigorous safety guidelines, optimal dosing regimens, and standardized administration procedures to ensure both efficacy and patient safety. Developing universally accepted clinical practice standards for the use of BVA in conventional medicine, especially for musculoskeletal and neurological disorders, will be crucial to promote its safe and evidence‐based integration into clinical care [62].

## 3. Conclusion

Bee venom is a complex mixture of chemicals with significant biological properties, which has been extensively studied and utilized in traditional medicine. The main constituents of BV are proteins and peptides, although other compounds are also present in smaller quantities. Among the components, melittin is the most abundant and well‐researched. Many studies have focused on the anti‐inflammatory and immunomodulatory actions of BV. However, to comprehensively understand the mechanisms of action of BV, more in vitro and in vivo investigations are required. Further research and clinical trials are warranted to refine the dosing protocols and ascertain the most appropriate duration of treatment for different pain conditions, thereby maximizing the benefits and minimizing potential risks associated with BVA therapy.

## Ethics Statement

The authors have nothing to report.

## Consent

The authors have nothing to report.

## Conflicts of Interest

The authors declare no conflicts of interest.

## Author Contributions

X.Z. and Y.D. drafted the manuscript. C.W. and H.L. were responsible for the conception and design of the study and led the project. H.Z. and A.A. conducted the primary investigation, secured the necessary resources, curated the data, and reviewed and revised the manuscript. S.H.A.E. and V.G.K. performed the formal statistical analysis, produced the figures and tables for the publication, and contributed to the review and editing of the manuscript. S.A.A., M.H.A.H.A, and Z.L. reviewed and edited the manuscript. Xiaodi Zou and Yanzhao Dong contributed equally to this work.

## Funding

No funding was received for this manuscript.

## Data Availability

Data sharing is not applicable to this article as no datasets were generated or analyzed during the current study.
